# A Systematic Review of Access to Rehabilitation for People with Disabilities in Low- and Middle-Income Countries

**DOI:** 10.3390/ijerph15102165

**Published:** 2018-10-02

**Authors:** Tess Bright, Sarah Wallace, Hannah Kuper

**Affiliations:** International Centre for Evidence in Disability, London School of Hygiene & Tropical Medicine, London WC1 E7HT, UK; Sarah.Wallace1@lshtm.ac.uk (S.W.); hannah.kuper@lshtm.ac.uk (H.K.)

**Keywords:** access, health care, rehabilitation, people with disabilities, low- and middle-income country, universal health coverage

## Abstract

Rehabilitation seeks to optimize functioning of people with impairments and includes a range of specific health services—diagnosis, treatment, surgery, assistive devices, and therapy. Evidence on access to rehabilitation services for people with disabilities in low- and middle-income countries (LMICs) is limited. A systematic review was conducted to examine this in depth. In February 2017, six databases were searched for studies measuring access to rehabilitation among people with disabilities in LMICs. Eligible measures of access to rehabilitation included: use of assistive devices, use of specialist health services, and adherence to treatment. Two reviewers independently screened titles, abstracts, and full texts. Data was extracted by one reviewer and checked by a second. Of 13,048 screened studies, 77 were eligible for inclusion. These covered a broad geographic area. 17% of studies measured access to hearing-specific services; 22% vision-specific; 31% physical impairment-specific; and 44% measured access to mental impairment-specific services. A further 35% measured access to services for any disability. A diverse range of measures of disability and access were used across studies making comparability difficult. However, there was some evidence that access to rehabilitation is low among people with disabilities. No clear patterns were seen in access by equity measures such as age, locality, socioeconomic status, or country income group due to the limited number of studies measuring these indicators, and the range of measures used. Access to rehabilitation services was highly variable and poorly measured within the studies in the review, but generally shown to be low. Far better metrics are needed, including through clinical assessment, before we have a true appreciation of the population level need for and coverage of these services.

## 1. Introduction

The World Health Organization (WHO) estimates that over one billion people, or 15% of the global population, live with a disability, with 80% living in low- and middle-income countries (LMICs) [1]. Disability, defined by the International Classification of Functioning, Disability and Health (ICF), is an umbrella term for impairments, activity limitations, and participation restrictions [2]. People with disabilities experience an impairment (e.g., visual impairment) because of a health condition (e.g., glaucoma). Contextual factors, both at the individual (e.g., age, sex) and wider societal level (e.g., access to health services, attitudes towards disability), play a crucial role an individual’s experience of the impairment.

People with disabilities often experience poorer levels of health than people without disabilities for various reasons [1]. By definition, people with disabilities have an underlying health condition which causes greater health needs. For example, people with chronic health conditions such as arthritis have regular ongoing health needs relating to the health condition and associated impairment [1]. People with disabilities may also be at risk of developing secondary health conditions such as depression [3]. Furthermore, evidence from a range of settings, both high-income countries and LMIC, suggests that people with disabilities face a multitude of barriers to accessing healthcare services. Poverty and disability are linked in a cycle, whereby poverty can lead to disability, and disability to poverty [4]; poverty and poor health are known to be linked through various mechanisms including though poorer living conditions, lifestyle factors (e.g., diet, smoking), and access to health services.

People with disabilities have a need to access the same general health care services as people without disabilities such as care-seeking when ill, vaccinations, and HIV treatment. In addition to general health services, people with disabilities also may require specific health care services related to their impairment, which includes rehabilitation. Rehabilitation is a broad term that encompasses a set of interventions to address impairments—activity limitations, and participation restrictions, as well as personal and environmental factors that have an impact on functioning [1]. Rehabilitation seeks to optimize functioning of people experiencing disabilities. Therefore, it includes the range of specific health services people with disabilities may require, from diagnosis, treatment, surgery, assistive devices, and therapy.

Evidence on access to rehabilitation services is sparse; however, there is expected to be very limited capacity to meet demand for these services in LMIC. The WHO estimates that there are less than ten skilled rehabilitation practitioners per 1 million population in LMIC [5]. Furthermore, the WHO estimates that between 5 and 15% of people in need for assistive devices in LMIC have received them [6]. Even fewer are expected to have hearing aids, with less than 3% of hearing aid need being met [7]. However, as is recognized in the WHO’s World Report on Disability, global data on unmet need for rehabilitation services is extremely sparse [1]. Unmet need for rehabilitation has a substantial impact on activity limitations, participation restrictions, and can result in poorer health and quality of life [1].

Rehabilitation has previously received little attention from governments, which has contributed to poor service availability and lack of co-ordination between services. Affordable and high-quality services should be available to all those in need. This is the main premise behind Universal Health Coverage (UHC), which is defined as, “ensuring all people have access to needed promotive, preventive, curative, rehabilitative, and palliative services they need, of sufficient quality to be effective, while ensuring that the use of these services does not expose the user to financial hardship” [8]. UHC is recognized as a key target in Goal 3 of the Sustainable Development Goals (SDGs) (Ensure healthy lives and promote well-being for all at all ages) [9], and so access to rehabilitation is essential in order to reach the SDG goals and targets. Access to rehabilitation for people with disabilities is also a human right, as stated in Article 26 of United Nations Convention for the Rights on People with Disabilities (UNCRPD) [10].

Recent global initiatives such as the Global Co-operative on Assistive Health Technology (GATE) strive for affordable and high-quality assistive technologies to be available for all those in need [11]. In February 2017, the WHO hosted a stakeholder meeting Rehabilitation 2030: A call to action, highlighting the issue of the substantial unmet need for rehabilitation around the world, and the lack of data on access to rehabilitation [5]. Considering the lack of data, we conducted a systematic review which aimed to summarize the current literature on access to rehabilitation for people with disabilities in LMIC, with a focus on health-related rehabilitation.

## 2. Materials and Methods

The systematic search was conducted in February 2017 for peer-reviewed articles that presented research findings on access to rehabilitation for people with disabilities in LMIC settings. The Preferred Reporting Items for Systematic Reviews and Meta-Analysis (PRISMA) statement was followed for conducting and reporting the review [1].

### 2.1. Eligibility Criteria

Studies were eligible if they met the following criteria: (1) quantitative research that included people with disabilities; (2) results reported access to rehabilitation for people with disabilities; and (3) research was undertaken in a LMIC as defined by the World Bank country classification 2017. No restrictions were placed on publication date, or language. Studies were excluded if the full text was not available after exhausting all possible sources. Duplicate reports from the same study were either combined if they reported different result or one was excluded if the results were the same.

### 2.2. Access to Rehabilitation Defined

For this review access was defined as use and coverage of services. Rehabilitation was defined in relation to the WHO definition as a “set of measures that assist individuals who experience or are likely to experience, disability to achieve and maintain optimal functioning in interaction with their environments” [1]. Using this definition, a broad range of interventions that may be required to maximize functioning were included: access to medical rehabilitation, access to therapy, coverage of assistive devices, and adherence to medication. Medical rehabilitation is defined as improving functioning through the diagnosis and treatment for health condition, reducing impairments and preventing or treating complications. Therapy is defined as restoring or compensating for loss of functioning, and preventing deterioration in functioning which may include physiotherapy, occupational therapy, and speech therapy. Assistive devices are defined as any equipment that is used to increase or maintain functional capabilities. We did not include studies measuring curative interventions, such as provision of spectacles, cataract surgery, hip replacement surgery, and similar treatments [12,13,14]. Whilst we recognize that rehabilitation extends beyond specialist health-related needs, this was beyond the scope of our review, which focused on health-related rehabilitation.

### 2.3. Types of Disability Measures

Studies defining disability using both the ICF definition (e.g., functioning, or activity limitations, and participation restrictions) and medical model definitions (i.e., specific impairments or disorders) were included.

### 2.4. Information Sources

Six databases (EMBASE, Global Health, CINAHL, Web of Science, MEDLINE, and PSYCINFO) were searched. The search strategy used key words for the following concepts: LMICs, people with disabilities, and access to health services. Terms were developed using MeSH or equivalent as well as from other reviews on similar topics. Boolean, truncation, and proximity operators were used to construct and combine searches for the key concepts as required for individual databases. An example of the search strategy is provided as Table S1. Systematic reviews identified through the search were reviewed for relevant included studies. If study protocols were identified, a search was made to determine whether the results of the study had been published. Furthermore, studies known to authors were included. No restrictions were made on language or time of publication.

### 2.5. Study Selection

All studies identified through the search process were exported to an EndNote database (version X7, Clarivate Analytics, Philadelphia, PA, USA) for removal of duplications and screening. Two reviewers (Tess Bright and Hannah Kuper) independently examined the titles, abstracts, and keywords of electronic records according to the eligibility criteria. Results were compared. The full texts were double screened (Tess Bright and Hannah Kuper) according to the eligibility criteria for final inclusion in the systematic review. Any disagreements in the selection of the full text for inclusion were resolved through discussion. 

### 2.6. Data Collection Process

Data were extracted in to a Microsoft Excel database developed for the purposes of this review. The first author (Tess Bright) extracted all data and this was independently examined by a second reviewer to ensure accuracy (Sarah Wallace). Data were extracted on the following study components:

- General study information, including author, year of publication
- Study design, sampling, and recruitment methods
- Study setting, and dates conducted
- Population characteristics including age, sex, and sample size
- Disability type/domain being studied, and means of assessing disability
- Results: main findings related to access to rehabilitation and any disaggregation by age, sex, urban-rural status, or other variables. We extracted data on the proportion covered by rehabilitation services in the population. Where unmet need was presented, we calculated the met need as one minus the unmet need.

We conducted a narrative synthesis due to the variation in included study designs, measurement of disability and outcomes which made meta-analysis impossible.

### 2.7. Risk of Bias in Individual Studies

Quality assessments of all eligible studies were carried out independently by two reviewers (Tess Bright and Sarah Wallace). We evaluated studies based on a set of criteria according to the SIGN50 guidelines [15]. Table 1 outlines the criteria used to evaluate studies.

## 3. Results

### 3.1. Study Selection

**Figure 1:**
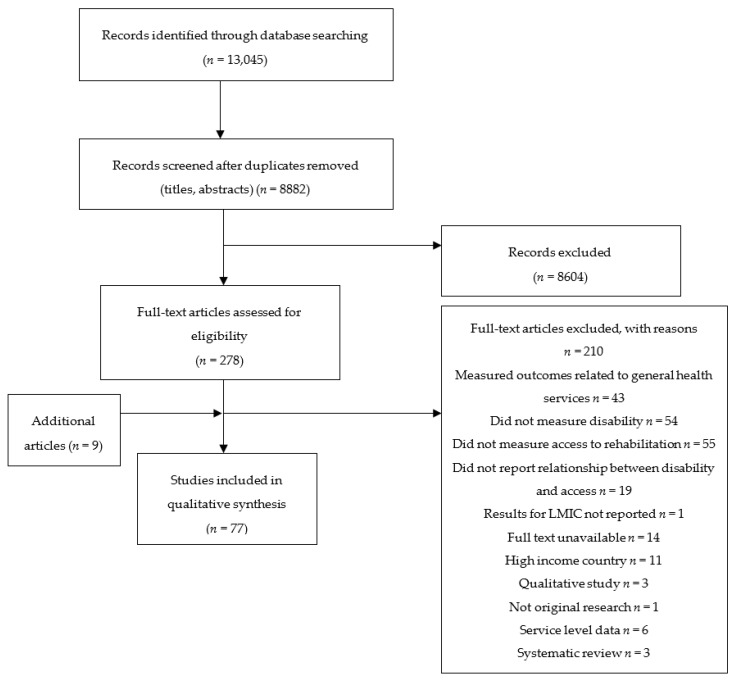
Flow chart of search results. (LMIC: Low- and Middle-Income Countries).

8886 unique records were identified through electronic searches. 8609 studies were excluded during title and abstract screen, resulting in 278 for the full text screen. Following full text review, 201 studies were excluded, and the full text could not be identified for 14 articles (Figure 1). Consequently, 77 studies were selected for inclusion and provided data for 106,462 people with disabilities across 64 countries.

### 3.2. Study Characteristics

Table 2 summarizes the characteristics of the studies eligible for inclusion. By region, most studies were conducted in sub-Saharan Africa (31%), followed by South Asia (18%), Latin America (16%), East Asia (16%), Middle East (9%), and Europe (3%). A further 8% were conducted in multiple countries. In terms of location, 49% were conducted in both urban and rural areas, with 18% in urban only and 13% in rural only (location unclear for 19% of studies). Most studies (73%) were conducted at subnational (e.g., district(s), or provincial level), with the remaining 27% carrying out national surveys. Over half of studies were conducted in 2010 or later (53%). The vast majority of studies were cross-sectional surveys (82%) with the remaining studies using cohort (5%), case control (10%) or retrospective longitudinal (3%) study designs. In terms of country income group, 33% of studies were conducted in low income, 28% in low-middle income, 29% in upper-middle income and 8% in countries of varying income levels.

### 3.3. Participants

Most studies included people of all ages (38%). 32% included adults only, 9% included older adults (>40 years), and 14% included children only (<18 years). In 6% of studies the age group was unclear. Considering disability domain, a large proportion of studies measured access outcomes related to mental impairment (44%), which we defined according to the International Classification of Diseases 10 (ICD10) “mental and behavioral disorders” included mental illnesses, intellectual impairment, and developmental delay. Epilepsy, although a neurological condition according to ICD10 was also grouped under mental impairment for simplicity. The remainder considered services related to hearing impairment (17%) visual impairment (22%), physical impairment (31%) or disability in general, across multiple domains (31%). The method of assessment of disability varied across studies, with 33 using self-reported measures (11 used the Washington Group short or extended set), 31 studies used clinical examination, four used a combination of reported and clinical measures, two used registry data, in two studies assessment methods were unclear, and the remaining three studies used alternative methods (e.g., community health worker report).

### 3.4. Outcome Types

Types of rehabilitation outcomes included:

- Medical rehabilitation: including received treatment/surgery, received diagnosis, access to, or ever received rehabilitation (any type), received therapy (physical, occupational, speech and language) (48 studies, 62%)
- Assistive devices: including hearing aids, mobility aids, low vision devices, or any assistive device (25 studies, 32%)
- Adherence: including adherence to treatment, treatment completion rate, and uptake of referral (25 studies, 32%)

In addition, data on barriers to accessing rehabilitation for people with disabilities were extracted as secondary outcomes in 23 studies (30%).

### 3.5. Description of Studies

Results of the 77 included studies are presented below by access to services specific to the following disability domains: hearing, mental health, physical, and visual. Where multiple domains were measured, and access outcomes were not disaggregated by domain, the results are presented in a separate section on rehabilitation for any disability.

#### 3.5.1. Access to Rehabilitation for Hearing Impairment

In total, 13 studies measured access to hearing specific services in 12 LMIC countries, and four World Bank regions. The study populations used to assess access varied across studies, with the majority using population-based data; however, one sampled children from deaf schools, two from registries and one from a clinic. Most studies in this group (seven studies) were conducted among people of all ages. Five studies were conducted in children, and two among older adults. The method of assessment varied, with five using the Washington Group short or extended set, one using the WHO ‘Ten Questions’, three using a bespoke self-reported tool, two conducting clinical assessments, and the remaining two using other methods (registry, community health worker identification). The access results are thus not directly comparable. Results are outlined in Table 3. Overall, nine studies measured coverage of assistive devices, seven studies measured access to medical rehabilitation, and one measured adherence. Coverage of assistive devices ranged from 0–66% across studies. General rehabilitation coverage (i.e., access to hearing services) was between 3–62%. Finally, one study measured adherence/compliance with referral and estimated this to be 34%.

Across studies, no clear patterns of access were seen by country group, locality, or by age. Coverage of assistive devices tended to increase with country income group but was typically quite low. One national study by Malta et al. (2016) in Brazil measured association between locality (urban or rural) and access and found a higher proportion had assistive devices in urban areas compared to rural areas. In terms of the quality of the evidence across studies, most studies were judged to have low risk of bias (eight studies). Six studies were judged to have high or medium risk of bias due to small sample size (three studies), means of assessing disability unreliable (three studies), or poor response rate (two studies).

#### 3.5.2. Access to Rehabilitation for Mental Impairment

In total, 34 studies measured access to specialist health services for people with mental impairments in 17 countries across six World Bank regions. Three studies were multi-country studies, for which it was possible to disaggregate results by country. For several countries, multiple studies were identified—three in China, three in Lebanon, four in Mexico, five in India, four in South Africa and four in Brazil. Considering age, the majority were conducted among adults (19 studies), among people of all ages, four among children, and one among older adults. Most studies sampled participants from the population (28 studies); the remaining sampled from schools (one study), clinic (three studies), or a variety of sources (two studies).

This category encompasses a broad range of conditions, from depression to intellectual impairment. Our search identified nine studies focusing on depression (or major depressive disorder), four studies on schizophrenia, three on epilepsy, five studies on psychiatric disorders, 14 measured general mental disorders with quite varied measures of assessment, two studies measured unspecified mental health conditions and the remaining two studies focused on intellectual impairment. In terms of method of assessment, a wide range of tools were used: five used a clinical diagnosis/examination, eight used the WHO composite international diagnostic interview, five used other validated questionnaires or tools (e.g., DSM-IV), two used the Washington Group short set, two used other validated self-reported tools, eight used bespoke self-reported tools (three of these combining with a clinical screen), one used household report, and one used global burden of disease data (see Table 4 for details).

In terms of outcomes, 28 measured access to medical rehabilitation, and five measured adherence to treatment. Access to medical rehabilitation for depression, which included treatment coverage and use of mental health services, most ranged from 0% for males in Mexico (subnational) to 54% in Brazil (national). El Sayed et al. (2015) found 65% of people with depression were in treatment across various LMIC using nationally representative data from the World Health Surveys. For schizophrenia, treatment coverage ranged from 50–71% in India (both subnational studies). Two multi-country studies were conducted, the first by Lora et al. (2012) found coverage of 11% (low income countries) to 31% (low-middle income countries) using the WHO Assessment Instrument for Mental Health Systems and the second by El Sayed et al. (2015) found coverage of 67% World Health Survey data. Coverage of epilepsy treatments ranged from 0% for older adults in Zimbabwe (subnational), to 52% among people of all ages in The Gambia (subnational). For children with intellectual disabilities coverage was higher: 73% in Ethiopia (subnational) and 87% in India (subnational) (two studies only). For other less specific conditions, coverage of medical rehabilitation ranged from 1% in China (national) (use of services, all ages) to 68% for adults in South Africa (subnational) (percent needing rehabilitation who received, all ages).

The broad range of conditions, source of participants, outcomes, and age groups mean that estimates within this group cannot be directly compared. However, it was clear that access for all outcomes was quite low across studies, except for children with intellectual impairments. There was considerable variation, even within studies conducted in the same country.

Across studies, no clear pattern was seen by country income level, locality or by age. One study by Lora et al. (2012) found lower treatment coverage in low income countries (11%) compared to low-middle income countries (31%). Considering other equity indicators, Li et al. (2013) and El Sayed et al. (2015) found higher coverage for insured people. Hailemariam et al. (2012) Andersson et al. (2013), Chikovani et al. (2015), Andrade et al. (2002) found no significant difference in access by employment, or income, while Ma et al. (2012) and Raban et al. (2010) found that poorer people were less likely to continue treatment. Demyttenaere et al. (2004) found an increase in coverage with severity of impairment in Colombia, Iraq, Lebanon, Mexico, Nigeria, and Ukraine, but not in other countries.

In terms of the quality of the evidence, the vast majority of studies included in this group were judged to have low risk of bias (30 studies). Three studies had high or medium risk of bias due to small sample size (three studies), unclear or low response rate (four studies), or unreliable means of assessing disability (five studies).

#### 3.5.3. Access to Rehabilitation for Physical Impairment

Table 5 provides the results of 24 studies measuring access to rehabilitation for physical impairment. Studies were conducted across 17 countries and five World Bank regions. Types of physical impairments were varied, including rheumatoid or other arthritis (five studies), cerebral palsy (two studies), leprosy (two studies), difficulties walking (six studies), amputation (one study), musculoskeletal impairment (three studies), and unspecified physical impairment (eight studies). In terms of method of assessment, four used the Washington Group short or extended set questions (self-reported difficulties walking), eight used other self-reported tools, one used a chronic disorders checklist, five used a clinical diagnosis, four selected participants from a registry, one used community health worker report, and one study the method was unclear. Five studies were conducted among adults, 11 among people of all ages, six among children and in two studies the age group was not presented. Outcomes included access to physical therapy, assistive devices, medical rehabilitation, and adherence. The vast majority of studies were conducted on population-based samples; however, six sampled from clinic/hospital, and two from registries.

Access results for arthritis varied, with the highest coverage seen in Jordan (subnational) (76%) and lowest in India (subnational) (4%). Adherence to leprosy treatment was also quite high (71–75% in Nepal and Chad, both subnational studies); however, this may reflect the fact that these were both clinic-based studies. Results were more varied for less specific physical impairments such as “difficulties walking”, musculoskeletal impairment, and physical impairment—with coverage of assistive devices ranging between 5–57% in Tanzania (subnational) and 41–93% in Cameroon (subnational) (depending on the type of assistive device). Coverage of medical rehabilitation in Brazil was 18%, while in South Africa this was 66%.

Coverage did not tend to increase with country income group or show a clear pattern by age or locality across studies. El Sayed et al. (2015) found higher coverage among those covered with insurance in a multi-country study [36].

Ten studies were judged to have low risk of bias. A further 14 studies were judged to have medium (ten studies) or high risk of bias (four studies) due to unclear or unreliable measure of disability or access (eight studies) or small sample size (four studies), or low response rate (three studies).

#### 3.5.4. Access to Rehabilitation for Vision Impairment

In total, 17 studies measured access to rehabilitation for people with visual impairment across 13 countries in four World Bank regions. Table 6 outlines the results of these studies. The method of assessment varied across studies with seven using self-reported tools (of these four used Washington Group), seven using clinical examination, and three using other methods (registry, community leaders).

Thirteen studies measured medical rehabilitation, five studies measured access to assistive devices, and one study measured uptake of referral. Medical rehabilitation for people with visual impairment included consultation with specialist provider, and surgery uptake. All but two studies used a population-based sample. Access to medical rehabilitation was varied, from 5% among people of all ages in Brazil (national) to 82% among people of all ages in Nigeria (subnational). Similarly, results for assistive device coverage were highly variable, but typically low.

Across studies, a clear pattern was not observed by country income group, age, or urban-rural status. Higher coverage was identified for people with higher levels of education in several studies; Kovai et al. (2007), Lee et al. (2013), Palyagi et al. (2008), but not all (Fletcher et al., 1999).

Considering the quality of studies in this category, 12 were judged as having low risk of bias. The remaining five studies had high or medium risk of bias due to low or unclear response rate (four studies), unclear measure of disability (two studies), or unclear measure of access (one study).

#### 3.5.5. Access to Rehabilitation for Any Disability

Table 7 provides the results of 28 studies measuring access to rehabilitation for any disability (i.e., those studies that did not disaggregate by impairment type, or reported overall coverage results). These studies were conducted in 23 countries in six regions: the majority in sub-Saharan Africa (12 studies). Outcomes included access to assistive devices (18 studies), general rehabilitation (22 studies), and adherence (one study). Most studies sampled participants from the population, with one each using clinic or registry as a sampling frame. 21 studies measured disability using self-reported tools, including 12 using the Washington Group questions, two using the Rapid Assessment of Disability tool, and the remainder used bespoke tools. Four studies used a clinical examination. Two studies used registries to identify participants.

Coverage of general rehabilitation varied across studies. Coverage was particularly low in India (subnational) and Bangladesh (subnational) at 5% and 7% respectively. In contrast studies in the Philippines, South Africa, Malaysia, and Brazil (all subnational studies) found higher coverage at 70%, 71%, 76%, and 80%. Substantial variation was also found for access to assistive devices, but generally coverage was low.

There did not appear to be a trend in coverage by country income group. The vast majority of these studies were conducted in both urban and rural areas and did not disaggregate results, thus examining patterns by locality was not possible. Furthermore, most studies were conducted among people of all ages, with no disaggregation of results by age group. Within studies, four studies examined coverage outcomes by indicators of equity. Three studies found lower coverage among females (Hosain et al. (1998), Eide et al. (2006), Eide et al. (2009)), but no consistent patterns by age, socioeconomic status or location were revealed.

Considering the strength of evidence for access to any specialist services, eight studies were judged to have high or medium risk of bias, while the remaining were assessed as having low risk. The main risks were—unclear or unreliable measure of disability (five studies), or low or unclear response rate (five studies).

#### 3.5.6. Barriers

Of the 77 included studies, 22 evaluated barriers to accessing rehabilitation as secondary outcomes. Commonly reported barriers included logistical factors (distance to service, lack or cost of transport), affordability (of services, treatment, lack of insurance), and knowledge and attitudinal factors (including perceived need, fear, and lack of awareness about the service) (Table 8). Many of these barriers identified are not unique to disability. However, particular barriers were disability-related, including discrimination from the health provider, provider lacking skills, and communication barriers, or potentially enhanced among people with disabilities (e.g., lack of affordability).

## 4. Discussion

### 4.1. Review of Findings

This systematic review summarises the available evidence on access to rehabilitation services for hearing (13 studies), visual (17 studies), physical (24 studies) mental (34 studies), and any disability-related service (27 studies). The review captured studies a wide range of World Bank geographic regions, and over 60 countries.

Access results were varied across studies. Access to hearing specific services ranged from 0 to 66%. For visual impairment this was 0 to 82%, physical 0 to 93%, mental 0 to 97% and any disability-related services was 5 to 80%. Despite the variation, overall, access was low; however, there were some outlier studies showing high coverage. The review highlighted that outcomes used to measure access to rehabilitation, as well as measures of impairment/disability, are varied making comparisons and generalizability difficult. Coverage of services where disability is measured using self-reported tools such as the Washington Group short set of functioning, assumes that people who report difficulties are in need of rehabilitation. This may not be the most accurate measure of coverage (e.g., people blind from cataract may require surgery, not low vision aids) and further work is required to develop standard methods of measurement. Most studies used population-based, cross-sectional data, where the population in need in a particular region were identified (i.e., a prevalence study) and asked about access to services. However, we included studies where participants were sampled from clinics, or registries. These studies are very likely to overestimate coverage given these individuals have already been in touch with some type of service.

In terms of barriers to accessing rehabilitation, common themes across 22 studies in a diverse range of settings included lack of affordability of services, equipment, or medication as reasons for not accessing care. In addition, logistical or geographical factors such as distance to the service, transportation problems, and a lack of a chaperone. Several service-related barriers including discrimination from provider, communication barriers, and lack of provider skill were also common. These barriers may be specific to or greater for people with disabilities than those without disabilities. Further research is needed to examine particular barriers to access that people with disabilities face in greater depth.

The quality of included studies was generally high. There was limited evidence to support an association of coverage with country income group, age, urban-rural location, or other variables such as socioeconomic status. Included studies did not routinely disaggregate results by these variables—with less than a third of studies measuring variables related to equity of coverage.

### 4.2. Consistency with Previous Reviews

To our knowledge, this is the first systematic review that has attempted to summarize the available evidence on access to health-related rehabilitation for people with disabilities in LMIC. Thus, there are few similar examples from the literature to which the results can be compared.

Several previous reviews have focused on coverage of mental health services, evidence on assistive device coverage, and rehabilitation workforce literature. In a recent scoping review by Matter et al. (2017), authors identified a lack of publications on assistive devices from LMIC, in particular with respect to data on hearing, communication or cognition [96]. Similarly, a previous review by De Silva et al. (2014) on coverage of mental health programs highlighted that there was limited evidence on the topic [97]. They noted coverage estimations varied across studies, making comparisons difficult and called for coverage estimates to be stratified by age, gender, socioeconomic status to understand equity of coverage. These conclusions align with the findings of our review.

Jesus et al. (2017) conducted a review of rehabilitation workforce literature [98]. They found that substantial shortages of rehabilitation workers are documented in low income countries, particularly in sub-Saharan Africa and Latin America—with only six physicians specialized in rehabilitation in sub-Saharan Africa. Few programs exist for obtaining a qualification in rehabilitation, with several studies reporting alternative health worker cadres which could mitigate this; however, there is limited evidence on effectiveness. Although these findings have a health systems perspective on access to health services, they help to explain the reported low coverage of rehabilitation services in many studies in our review. Bruckner et al. (2010) also found that out of 58 LMIC involved in the WHO Assessment Instrument for Mental Health Systems surveys, that the vast majority did not meet expected health workforce targets for delivery of mental health services [99].

Several national surveys have been conducted in high-income countries such as the United Kingdom, the United States, and Korea. In the United States, a nationwide survey of people with cerebral palsy, multiple sclerosis, and spinal cord injury found that nearly one third of those who indicated a need did not receive assistive equipment every time it was needed. Over half of people had an unmet need for rehabilitative services [100]. In Korea, a 2009 nationally representative study (Korean National Health and Nutrition Examination Survey—KHANES) found that less than 10% of people with depressive mood had used mental health services [101]. In the United Kingdom, analysis of the European Health Interview Survey found that people with severe disability had higher odds of facing unmet need for health care, with the largest gap for mental health care [102]. Although these studies show high unmet need for services also exists in high-income contexts, access to rehabilitation is likely to be much poorer in LMIC.

The WHO have commonly cited statistics on coverage of assistive devices. For instance, it is estimated that hearing aid production meets less than 10% of the global need and less than 3% of people who need hearing aids in LMIC actually receive them. Furthermore, previous WHO estimates suggests that in many LMIC, 5–15% of people with disabilities have access to assistive devices [6]. Our review found wide variation in coverage of hearing aids and assistive devices but does agree that coverage is generally low. Again, the range of measurements of both disability and access limit comparability across studies.

### 4.3. Implications for Practice

This review has shown that in general, access to rehabilitation services is low in many LMIC. However, evidence is lacking from many countries of the world. To enable full implementation of the UNCRPD, member states must ensure that rehabilitation services are accessible to people with disabilities. Despite the UNCRPD providing a clear legal and regulatory framework, this review alongside key publications from the WHO, suggests that people with disabilities are not receiving a range of specific health services required to improve functioning. Evidence suggests that per capita income is linked to the level of implementation of the UNCRPD—underlining the major challenge for LMIC [103]. As outlined in the call to action in Rehabilitation 2030 there is an urgent need to address the unmet need for these services [5]. Although we have specifically focused on people with disabilities, rehabilitation has a broader scope, with some people needing rehabilitation temporarily at certain points in life (e.g., after a sports injury). Thus, addressing rehabilitation needs for people with disabilities has a wider benefit. Increasing life expectancy means the needs for rehabilitation will also increase, reinforcing the need to address this gap.

Rehabilitation should be integrated in to health systems at all levels to maximize access and achieve UHC. *Rehabilitation in Health Systems* guidance from the WHO provides recommendations for member states to strengthen and expand the availability of quality rehabilitation [104]. These, and other initiatives, include supply-side interventions, which attempt to address the dearth of services available to provide rehabilitation in LMIC. For instance, the GATE program of the WHO aims to improve access to affordable devices globally through various mechanisms [11]. Community-based models of health care delivery have been attempted for specific health services including: mental health, eye care, and ear and hearing care. These task shifting approaches are endorsed by the WHO as a mechanism to overcome skills shortages and reach underserved populations [105]. Telemedicine is a growing area for provision of rehabilitation and may help overcome the geographical barriers commonly reported in the literature. As an example, in the field of hearing impairment, telemedicine has been used for screening, diagnosis, and hearing aid fittings [106]. Furthermore, mobile technology has huge potential for improving access to rehabilitation. For example, in Kenya smartphone-based assistive technologies have been tested for students with visual impairment with positive impact on access to education, and participation in everyday life [107]. Sureshkumar et al. (2015) have tested a smartphone-based educational intervention for people with physical impairments following stroke in India [108].

Furthermore, demand-side interventions such as financial incentives and health promotion/education may help to improve uptake of available services. This includes strategies such as ensuring health insurance covers rehabilitation services, which will help to avoid catastrophic health expenditure. Two systematic reviews conducted by Bright et al. found that delivery of services at or close to home, text-message reminders, and vouchers may be beneficial for improving access to services for children in LMIC, but more evidence is needed on “what works” to improve access for people with disabilities [109,110].

### 4.4. Implications for Research

#### Use Common Definitions of Disability and Coverage

To monitor progress towards the SDGs with respect to disability, and for program-planning purposes, key indicators of access to and coverage of rehabilitation should be developed, with a uniform method of measurement to allow comparability. This includes using clear definitions of what is meant by rehabilitation (e.g., medical rehabilitation, assistive technology, and therapy) and how coverage or access are measured. Access to health-related rehabilitation in this review was usually measured in terms of “coverage”, that is the proportion of people needing a service who reported receiving it. However, this may overestimate coverage as the service may be inadequate and/or the full course of treatment may not be completed. Better measures of “access” are therefore needed. Furthermore, common definitions of disability should be adopted. Ideally, this should focus on clinical measurement of impairment, as these will also provide further information about the rehabilitation needs [111]. For instance, self-reported hearing difficulties does not give adequate information about service needs, which may range from basic wax removal to more complex surgeries or hearing aid fitting. Clinical assessment would provide the information needed to plan rehabilitation and specialist services. In addition, equity of service coverage should be assessed as part of any data collection to monitor access to rehabilitation. Sociodemographic information such as age, gender, socioeconomic status, locality, should be collected which can then allow data disaggregation. Monitoring the effectiveness and quality of rehabilitation care received is crucial for informing service delivery improvements, and ensuring functioning is maximized for people with disabilities.

### 4.5. Limitations and Strengths

This review has several limitations that need to be taken in to account. We focused on literature from peer-reviewed sources, and it is possible that some relevant data is available in grey literature sources, not captured in our search. Although we placed no restrictions on language, the electronic searches were conducted on six databases in the English language, and thus some literature may have been missed. Although our review encompassed a broad range of countries, and all the World Bank regions except for North America (high income), a third of studies came from sub-Saharan Africa. Our results may be slightly biased towards the conditions in these countries. However, the range of countries in sub-Saharan Africa included were limited to 15 of the 48 countries—suggesting that despite the largest proportion of data coming from this region, further research is required. Data was lacking from many parts of the world, with only 16% of included studies from Latin American countries, therefore included studies may not be representative of the level of access to rehabilitation in many LMICs. Studies may have been conducted in countries where stronger rehabilitation services exist, which may exaggerate the results found. The vast majority of studies were conducted at district level (73%), rather than national level, so making inferences about the situation of rehabilitation access in a whole country is limited. In the analysis we compared results by country income level (low, low-middle, and upper-middle). Ideally, a comparison between the results of studies by region (e.g., LMICs in Africa) would have been made, however the range of measurement types used limits comparability. Our review did not have a focus on the availability of services, which is an important dimension of access and may help to explain poor coverage of rehabilitation [112]. The scope of our review was on health-related rehabilitation and does not focus on broader needs such as education or work-related rehabilitation. We also did not include access to sign language education, rather than medical interventions for hearing impairment. Thus, we have not captured access to rehabilitation in its broadest sense as defined in Rehabilitation 2030. This warrants further attention. We did not assess the costs of accessing rehabilitation services, even though financial constraints were a major reason for not seeking care. Finally, we did not place any restrictions on publication date in our review, which means we have captured available literature to date; however, some studies may be outdated, and not reflective of the current level of access in the country studied.

There are also several strengths. This review was large, and adopted a systematic approach, following Cochrane guidelines. We used a comprehensive list of search terms to capture the literature available on this topic. It captured a broad range of disability types, and across a diverse range of countries and published in different languages.

## 5. Conclusions

This systematic review on access to rehabilitation for people with disabilities found wide variation in reported coverage across studies. In general, coverage appeared to be low for medical rehabilitation, assistive devices, therapy, and adherence. However, the review has identified a need to develop standard indicators for measuring coverage of rehabilitation to allow comparability. There is also a need to use comparable measures of disability. Common measures will contribute towards a greater understanding of the met and unmet needs for rehabilitation for people with disabilities and allow planning of appropriate services.

## Figures and Tables

**Table 1:** Quality assessment criteria and ratings.

**Assessment Criteria**	
Study design, sampling method is appropriate to the study question Adequate sample size (>100 participants), or sample size calculations undertaken Response rate reported and acceptable (>70%) Disability/impairment measure is clearly defined and reliable Measure of access clearly defined and reliable Potential confounders taken into account in analysis (if necessary) Confidence intervals are presented	
**Overall Ratings**	
++	Low risk of bias: All or almost of the above criteria were fulfilled, and those that were not fulfilled were thought unlikely to alter the conclusions of the study
+	Medium risk of bias: Some of the above criteria were fulfilled, and those not fulfilled were thought unlikely to alter the conclusions of the study
−−	High risk of bias: Few or no criteria were fulfilled, and the conclusions of the study were thought likely or very likely to alter with their inclusion

**Table 2:** Characteristics of included studies.

Variable	Number	%
Region		
Latin America/Caribbean	12	16%
East Asia/Pacific	12	16%
Sub-Saharan Africa	24	31%
Middle east	7	9%
South Asia	14	18%
Europe/Central Asia	2	3%
Various	6	8%
Country income group		
Low	26	33%
Low-middle	22	28%
Upper-middle	23	29%
Various	6	8%
Location		
Urban	14	18%
Rural	10	13%
Both	38	49%
Unclear	15	19%
Decade of publication		
1990–1999	11	14%
2000–2009	25	32%
2010–current	41	53%
Age of participants		
All ages	29	38%
Adults only	25	32%
Older adults	7	9%
Children only	11	14%
Unclear age/not presented	5	6%
Study design		
Cross-sectional	63	82%
Retrospective longitudinal study	2	3%
Case control study	8	10%
Cohort	4	5%
Disability domain		
Hearing	13	17%
Vision	17	22%
Physical	24	31%
Mental	34	44%
Any disability	27	35%
Multiple domains	29	38%

**Table 3:** Access to hearing impairment specific services (D = disability).

Study Author, Year	Country (Study Location)	World Bank Region	Country Income Group	Locality (Urban or Rural)	Study Type	Participant Source	N (%D)	Age	Means of Assessing Disability	Outcome	Proportion Covered by Type of Rehabilitation (%)			Risk of Bias
											Medical Rehabilitation	Assistive Devices	Adherence to Treatment	
Allain et al. (1997) [16]	Zimbabwe (Bindura, Marondera)	SSA	Low income	Both	Cross-sectional study	Population	278 (NS); 55 (20%) with hearing impairment	Older adults	Self-report (bespoke tool, but unclear method) and observation by nurses	Wearing hearing aids when needed	-	0	-	Medium: adequate sample size, but small number with hearing loss, and unclear how hearing loss assessed
Bernabe-Ortiz et al. (2016) [17]	Peru (Morropon)	SSA	Upper-middle income	Semi-urban	Case control study	Population	322 (50%)	All ages	Washington Group short set	Coverage of hearing aids (proportion of those who use hearing aids among those reported in need)	-	9	-	Medium: low response rate
Danquah et al. (2015) [18]	Haiti (Port-au-Prince)	LA	Low income	Urban	Case control study	Population	356 (50%)	All ages	Washington Group short set	Met need for medical rehabilitation	3	3	-	Low
Devendra et al. (2013) [19]	Malawi (Lilongwe)	SSA	Low income	Unclear	Case control study	Clinic	592 (50%)	Children	WHO ten questions	Proportion of children who attended ear clinic of those in need	14	-	-	Low
Kuper et al. (2016) [20]	Tanzania (Mbeya, Tanga, Lindi)	SSA	Low income	Both	Case control study	Population	807 (39%)	All ages	Washington Group short set	Coverage of hearing aids (proportion of those who use hearing aids among those reported in need)	-	0	-	Low
Maart et al. (2013) [21]	South Africa (Cape Town)	SSA	Upper-middle income	Urban	Cross-sectional study	Population	151 (100%)	All ages	Washington Group short set	% needing hearing therapy that received	42	-	-	Low
Mactaggart et al. (2015) [22]	Cameroon (Fundong Health District)	SSA	Low-middle income	Unclear	Case control study	Population	845 (60%)	All ages	Washington Group extended set and clinical assessment	Coverage of hearing aids	-	24	-	Low
	India (Mahbubnagar)	SA	Low-middle income				703 (61%)				-	6	-	Low
Malta et al. (2016) [23]	Brazil (National)	LA	Upper-middle income	Both	Cross-sectional study	Population	204,000 (NS)	All ages	Self-report (bespoke tool)	Attendance at rehabilitation services for those in need	8 (9 urban, 4 rural)	-	-	Low
Nesbitt et al. (2012) [24]	Bangladesh (Natore, Sirajgani)	SA	Low income	Both	Prospective cohort study	Population	1308 (100%)	Children	Clinical assessment	Uptake/compliance with referral for assistive device, therapy, further investigation, medicine, or surgery	-	-	34	Low
Omondi et al. (2007) [25]	Kenya (Kisumu)	SSA	Low income	Both	Cross-sectional study	Deaf schools	33 (100%)	Children	Clinical assessment	Visit for hearing assessment (diagnosis); hearing aid use (assistive device)	27	0	-	High: small sample size
Padmamohan et al. (2009) [26]	India (Kerala)	SA	Low-middle income	Rural	Cross-sectional study	Population	98 (100%)	Children	Households of children with disabilities were identified with community health workers	Use of rehabilitation treatment	16	-	-	Medium: small sample size; unclear measure of disability
Ribas et al. (2015) [27]	Brazil (Curibita)	LA	Upper-middle income	Rural	Cross-sectional study	Clinic	578 (32%)	Older adults	Self-report (bespoke tool)	Had hearing test (diagnosis); wore hearing aids (assistive device)	28	16	-	Low: unreliable measure of disability
Tan et al. (2015) [28]	Malaysia (Penang)	EAP	Upper-middle income	Unclear	Cross-sectional study	Registry	305 (100%)	Children	Registry	Coverage of hearing aids (assistive devices); proportion accessing hearing services)	62	66	-	High: poor response rate, and unreliable measure of disability

SSA: sub-Saharan Africa, LA: Latin America, SA: South Asia, EAP: East Asia & Pacific.

**Table 4:** Results for studies measuring mental impairments (D = disability).

Study Author, Year	Country (Study Location)	World Bank Region	Country Income	Locality (Urban/Rural)	Study Type	Participant Source	N (%D)	Age Group	Specific Condition	Method of Assessment	Outcome	Proportion Covered by Rehabilitation Type %		Risk of Bias
												Medical Rehabilitation	Adherence to Treatment	
Studies measuring mental health and psychiatric disorders														
Abas et al. (1997) [29]	Zimbabwe (Harare)	SSA	Low income	Urban	Cross-sectional study	Population	51 (100%)	Adults	Depression and anxiety	Screening questionnaire and clinical examination	Receipt of antidepressant or anxiolytic	0 (antidepressant) 10 (anxiolytic)	-	Medium: small sample size
Alekhya et al. (2015) [30]	India (Andhra Pradesh)	SA	Low-middle	Both	Cross-sectional study	Clinic	103 (100%)	Adults	Depression	Clinical diagnosis	Proportion with good adherence	-	30	Medium: unclear measure of disability
Andersson et al. (2013) [31]	South Africa (Eastern Cape)	SSA	Upper-middle	Both	Cross-sectional study	Population	977 (31%)	Adults	Depression	DSM-IV schedule (mini international neuropsychiatric review)	Proportion of those emotionally troubled who sought care	43	-	Low
Hailemariam et al. (2012) [32]	Ethiopia (9 regions)	SSA	Low income	Both	Cross-sectional survey	Population	449 (100%)	Adults	Depression	World Mental Health Survey version of the Composite International Diagnostic Interview	Visiting health facilities for depressive episodes	23	-	Low
Snyder et al. (1999) [33]	Mexico (Jalisco)	LA	Upper-middle	Rural	Cross-sectional study	Population	945 (6.2%)	Adults	Depression	WHO World Mental Health Composite International Diagnostic Interview	Treatment received	Male 0; Female 13.0	-	Low
Karam et al. (1994) [34]	Lebanon (Bejjeh, Kornet Shehwan, Ashrafieh, Ain Remmaneh)	ME	Upper-middle	Unclear	Cross-sectional study	Population	213 (100%)	Adults	Major depressive disorder	Diagnostic Interview Schedule (DIS) by psychologists	Consulted doctor; consulted other professional; treatment received	23; 6; 30	-	Medium: risk of recall bias
Fujii et al. (2012) [35]	Brazil (National)	LA	Upper-middle	Both	Cross-sectional, web-based survey	Population (identified through the web)	9789 (10%)	Adults	Major depressive disorder	Self-report (bespoke tool) followed by validated questionnaire	Currently taking prescription medication	54	-	High: risk of selection bias
El Sayed et al. (2015) [36]	48 LMICs (various National level surveys)	Various	Various	Both	Cross-sectional study (World Health Surveys)	Population	197,914 (NS)	Adults	Depression and schizophrenia	Self-report (bespoke tool)	Proportion in treatment: depression, schizophrenia	65; 67	-	Low
Raban et al. (2010) [37]	India (Assam, Karnataka, Maharashtra, Rajasthan, Uttar Pradesh, West Bengal)	SA	Low-middle	Both	Cross-sectional study	Population	9994 (NS)	Adults	Depression and schizophrenia	Self-report (validated tool)	Treatment coverage: depression; schizophrenia	12; 50	-	Medium: means of assessing disability not reliable
Padmavathi et al. (1998) [38]	India (Madras)	SA	Low income	Urban	Cross-sectional study	Population	261 (100%)	All ages	Schizophrenia	Family report using screening tool, and detailed examination by a psychiatrist	Ever received treatment	71	-	Low
Lora et al. (2012) [39]	50 LMICs (National)	Various	Various	Unclear	Cross-sectional survey	Various	Unclear	Adults	Schizophrenia	Global burden of disease data for prevalence of schizophrenia, and number of people who received care (facility level data)	Treatment coverage (psychiatrist, mental health professionals)	11 (Low income); 31 (Low-middle income)	-	Low
Demyttenaere et al. (2004) [40]	China (National)	EAP	Low-middle	Urban	Cross-sectional study	Population	1628 (21%)	Adults	Mental disorders	WHO composite international diagnostic interview (WMH, CIDI)	Sought treatment for condition in the past 12 months: mild; moderate; serious	Beijing: mild 2; serious: 12 Shanghai: serious: 0.5	-	Low
	Nigeria (National)	SSA	Low income	Urban			1682 (14%)					10	-	Low
	Ukraine (National)	EU	Low-middle	Both			1720 (56%)					Mild 7 Moderate 17 Serious 19	-	Low
	Lebanon (National)	ME	Upper-middle	Both			1029 (47%)					Mild 4.5 Moderate 10 Serious 15	-	Low
	Colombia (National)	LA	Low-middle	Urban			2442 (33%)					Mild 8 Moderate 12 Serious 24	-	Low
	Mexico (National)	LA	Upper-middle	Urban			2362 (30%)					Mild 10 Moderate 19 Serious 20	-	Low
Andrade et al. (2002) [41]	Brazil (Sao Paulo)	LA	Upper-middle	Urban	Case control study	Population	1464 (27%)	Adults	Mental disorders	WHO World Mental Health Composite International Diagnostic Interview	Received specialty medical care: any disorder; mood; anxiety; substance use	13; 23; 20; 10	-	Low
Caraveo et al. (1999) [42]	Mexico (Mexico City)	LA	Upper-middle	Urban	Cross-sectional study	Population	1937 (8.3%)	Adults	Mental health condition	WHO World Mental Health Composite International Diagnostic Interview	Care seeking for mental health condition	Total proportion seeking help < 50%	-	Medium: response rate lower than 70%
Loeb et al. (2004) [43]	Malawi (National)	SSA	Low income	Both	Cross-sectional study	Population	1574 (100%)	All ages	Mental/emotional difficulties	Self-report (bespoke tool)	Ever received rehabilitation (medical)	22	-	Low
Eide et al. (2006) [44]	Zambia (National)	SSA	Low income	Both	Cross-sectional study	Population	2865 (100%)	All ages	Difficulties remembering, concentrating	Washington Group short set	Ever received rehabilitation (medical)	30	-	Low
Alhasnawi et al. (2009) [45]	Iraq (National)	ME	Low-middle	Both	Cross-sectional study	Population	4332 (14.5%)	Adults	Mental disorders	Questionnaire based on ICD10 and DSM-IV	Any health care treatment (mild; moderate; serious)	3; 4; 17	-	Low
Li et al. (2013) [46]	China (National)	EAP	Upper-middle	Both	Cross-sectional study	Population	2.6 million (0.6%)	All ages	Mental disorders	Self-report (bespoke tool) followed by clinical examination and WHO DAS	Use of services: rehabilitation; medication	1; 40	-	Low
Maart et al. (2013) [21]	South Africa (Cape Town)	SSA	Upper-middle	Urban	Cross-sectional study	Population	151 (100%)	All ages	Difficulties remembering	Washington Group short set	Proportion needing treatment who received	68	-	Low
Malta et al. (2016) [23]	Brazil (National)	LA	Upper-middle	Both	Cross-sectional study	Population	20,400 (6%)	All ages	Mental impairment (unspecified)	Self-report (bespoke tool)	Attendance at rehabilitation services	30	-	Low
Chikovani et al. (2015) [47]	Georgia (conflict affected areas)	EU	Upper-middle	Unclear	Cross-sectional study	Population (conflict affected areas)	3600 (30%)	Adults	Mental impairment	Self-report (bespoke) and validated clinical tools	Self-reported problem and sought care	39	-	Low
Trump et al. (2006) [48]	South Africa (National)	SSA	Upper-middle	Both	Cross-sectional study	Support group members, leaders	331 (100%)	All ages	Mental disorders	Self-report (bespoke tool)	Compliance (self-report)	-	32	High: low response rate, means of assessing disability unreliable
Ormel et al. (2008) [49]	6 LMICs (regional: Colombia, Mexico, China; national: Lebanon, South Africa, Ukraine)	Various	Various	Both	Cross-sectional study	Population	73,441 (NS)	Adults	Mental disorders	Self-report (Chronic disorders checklist)	Treatment prevalence by type of impairment: mental disorders (visiting a professional)	8	-	Low
Seedat et al. (2009) [50]	South Africa (National)	SSA	Low-middle	Both	Cross-sectional study	Population	4317 (NS)	Adults	Mental disorders	World Health Organization (WHO) Composite International Diagnostic Interview	Sought treatment for condition in the past 12 months	25	-	Low
Ma et al. (2012) [51]	China (Guangdong)	EAP	Upper-middle	Urban	Cohort study	Population, hospitals	1386 (100%)	Adults	Psychiatric disorders	Clinical diagnosis	Adherence to medication	-	95	Low
Caraveo et al. (1997) [52]	Mexico (Mexico City)	LA	Upper-middle	Urban	Cross-sectional study	Population	2857 (28.7%)	All ages	Psychiatric disorders	WHO World Mental Health Composite International Diagnostic Interview	Care seeking for mental health condition	14	-	Medium: response rate lower than 70%
Paula et al. (2014) [53]	Brazil (North, Northeast, Central, Southeast)	LA	Upper-middle	Both	Cross-sectional study	Schools	1721 (12%)	Children	Psychiatric disorders	Validated tool (KSADS-PL) based on caregiver report	Mental health service use in past 12 months: affective; anxiety; disruptive; eating; psychotic disorder; co-morbidity	20; 17; 20; 9; 0; 30	-	Low
Chadda et al. (2000) [54]	India (Delhi)	SA	Low income	Not clear	Retrospective study	Clinic	80 (100%)	All ages	Psychiatric morbidity (schizophrenia, bipolar, unspecified psychosis)	Clinical diagnosis	Compliance with treatment regimen	-	97	High: small sample size
Llosa et al. (2014) [55]	Lebanon (Burj el-Barajneh refugee camp)	ME	Upper-middle	Urban	Cross-sectional study	Population	194 (45%)	Adults	Psychiatric disorders	WHO UNHCR Assessment Schedule of Serious Symptoms in Humanitarian Settings (WASSS), followed by clinical exam	Treatment coverage (received psychological or psychiatric care)	6	-	Medium: Low response rate
Results of studies measuring intellectual impairment														
Padmamohan et al. (2009) [26]	India (Kerala)	SA	Low-middle	Rural	Cross-sectional study	Population	98 (100%)	Children	Intellectual impairment	Households of children with disabilities were identified by community health workers	Treatment received	87	-	Medium: small sample size; unclear measure of disability
Dejene et al. (2016) [56]	Ethiopia (Addis Ababa)	SSA	Low income	Urban	Cross-sectional study	Clinic	102 (100%)	Children	Intellectual disability, autism spectrum disorder	Clinical diagnosis	Met need for treatment by health professional	73 *	-	Low
Results of studies measuring epilepsy														
Allain et al. (1997) [16]	Zimbabwe (Uzumba Maramba Pfungwe, Bindura, Marondera)	SSA	Low income	Both	Cross-sectional study	Population	278 (NS)	Older adults	Epilepsy	Self-report (bespoke tool, method unclear), nurse observation	Receipt of anti-epileptic medication	0	-	Medium: unclear measure of disability
Coleman et al. (2002) [57]	Gambia (Farafenni)	SSA	Low income	Rural	Cross-sectional study	Population	69 (100%)	All ages	Epilepsy	Screening questionnaire followed by psychologist review	Ever sought biomedical treatment for epilepsy (medication)	52	-	Low
Nesbitt et al. (2012) [24]	Bangladesh (Natore, Sirajgani)	SA	Low income	Both	Key informant method; prospective cohort study	Population	1308 (100%)	Children	Epilepsy	Clinical diagnosis	Took up referral	-	34	Low

* Met need calculated as 100-unmet need (27.5% unmet need for treatment by health professional). SSA: sub-Saharan Africa, LA: Latin America, SA: South Asia, EAP: East Asia & Pacific, ME: Middle East; EU: Europe.

**Table 5:** Results for physical impairment.

Study Author, Year	Country (Study Location)	World Bank Region	Country Income	Locality (Urban/Rural)	Age Group	Study Type	Participant Source	N (%D)	Specific Condition	Method of Assessment	Outcome	Proportion Covered by Type of Rehabilitation %			Risk of Bias
												Medical Rehabilitation	Assistive Device	Adherence	
Bernabe-Ortiz et al. (2016) [17]	Peru (Moroppan)	LA	Upper-middle	Semi-urban	All ages	Case control study	Population	798, 308 (5%)	Difficulties walking (WG)	Washington Group short set	Coverage: Walking stick; wheelchair, crutches, standing frame	-	26; 33; 26; 10	-	Medium: low response rate
Bigelow et al. (2004) [58]	Haiti (Port-de-Paix, Cap-Haitien, Fort Liberte, Port-au-Prince, Jacmel, Les Cayes, Jeremie)	LA	Low income	Both	All ages	Cross-sectional study	Registry, hospitals, organizations	164 (100%)	Amputation	Registry, hospitals, word of mouth	Had a prosthetic limb in the past, or currently had	-	25	-	High: small sample size
Devendra et al. (2013) [19]	Malawi (Lilongwe)	SSA	Low income	Unclear	Children	Case control study	Clinic	592 (50%)	Physical impairment (unspecified)	WHO ten questions	Proportion of children who attended physiotherapy	42			Low
Doocy et al. (2016) [59]	Jordan (National)	ME	Upper-middle	Both	Not presented	Cross-sectional study	Population	9580 (14%)	Arthritis	Self-report (bespoke tool)	Care sought for chronic condition	76	-	-	Medium: unreliable measure of disability
El Sayed et al. (2015) [36]	48 LMIC (National)	Various	Various	Both	Adults	Cross-sectional study	Population	197,914 (NS)	Arthritis	Self-report (bespoke tool)	Proportion in treatment	77	-	-	Low
Eide et al. (2006) [44]	Zambia (National)	SSA	Low income	Both	All ages	Cross-sectional study	Population	2865 (100%)	Difficulties walking (WG)	Self-report (bespoke tool)	Ever received assistive devices; Ever received rehabilitation (medical)	25	50	-	Low
Gadallah et al. (2015) [60]	Egypt (Cairo)	ME	Low-middle income	Urban	Adults	Cross-sectional study	Clinic	140 (100%)	Arthritis (rheumatoid)	Patients registered with rheumatology clinic	Medication adherence test	-	-	0	High: unclear measure of disability; clinic-based sample; recall bias likely
Kumar et al. (2004) [61]	Nepal (Dhanusa)	SA	Low income	Unclear	Adults	Cross-sectional study	Clinic	273 (42%)	Leprosy	Clinical examination (WHO guidelines)	Treatment completion	-	-	71	Medium: unclear how patients selected, clinic-based sample
Kuper et al. (2016) [20]	Tanzania (Mbeya, Tanga, Lindi)	SSA	Low income	Both	All ages	Case control study	Population	254 (50%)	Difficulties walking (WG)	Washington Group short set + albinism	Coverage of: Wheelchair; crutches; walking stick; standing frame	-	5; 50; 53; 57	-	Low
Loeb et al. (2004) [43]	Malawi (National)	SSA	Low income	Both	All ages	Cross-sectional study	Population	1574 (100%)	Difficulties walking (WG)	Self-report (bespoke tool)	Ever received assistive devices; Ever received rehabilitation (medical)	31	25	-	Low
Malta et al. (2016) [23]	Brazil (National)	LA	Upper-middle	Both	All ages	Cross-sectional study	Population	204,000 (NS)	Physical impairment (unspecified)	Self-report (bespoke tool)	Attendance at rehabilitation services	18	-	-	Low
Maart et al. (2013) [21]	South Africa (Cape Town)	SSA	Upper-middle	Urban	All ages	Cross-sectional study	Population	151 (100%)	Difficulties walking (WG)	Washington Group short set	Medical rehabilitation coverage	66	-	-	Low
Mactaggart et al. (2015) [22]	India (Mahbabnagar)	SA	Low-middle income	Unclear	All ages	Case control study	Population	845 (60%)	Difficulties walking (WG)	Washington Group extended set	Coverage of: Wheelchair; crutches; walking stick; standing frame	-	26; 43; 87; 58	-	Low
	Cameroon (Fundong Health District)	SSA	Low-middle income					703 (61%)					41; 32; 93; 33		
McConachie et al. (2000) [62]	Bangladesh (location unclear)	SA	Low income	Both	Children	Cohort study	Clinic	47 (100%)	Cerebral Palsy	Clinical diagnosis	Attendance at 8–9 distance training package sessions	-		29	Medium: small sample size
Nesbitt et al. (2012) [24]	Bangladesh (Natore, Sirajgani)	SA	Low income	Both	Children	Cross-sectional study	Population	1308 (100%)	Physical impairment (unspecified)	Clinical assessment	Took up referral	-	-	50	Low
Ormel et al. (2008) [49]	Various (National)	Various	Various	Both	Not presented	Cross-sectional study	Population	73,441 (NS)	Musculoskeletal impairment (MSI)	Chronic disorders checklist	Treatment prevalence	52	-	-	Low
Padmamohan et al. (2009) [26]	India (Kerala)	SA	Low-middle income	Rural	Children	Cross-sectional study	Population	98 (100%)	Physical impairment (unspecified)	Community health workers assessment	Treatment received	47	-	-	Medium: small sample size; unclear measure of disability
Raban et al. (2010) [37]	India (Assam, Karnataka, Maharashtra, Rajasthan, Uttar Pradesh, West Bengal)	SA	Low-middle income	Both	Adults	Retrospective study	Population	9994 (NS)	Arthritis	Self-report (validated)	Treatment coverage	58	-	-	Medium: unreliable measure of disability
Saleh et al. (2015) [63]	Jordan (Amman)	ME	Upper-middle	Both	Children	Cross-sectional study	Clinic	116 (100%)	Cerebral palsy	Clinical diagnosis	Proportion who received treatment for a range of problems	Range: 24–100% (median: 50%)	-	-	High: unclear response rate; small sample size; selection bias
Schafer et al. (1998) [64]	Chad (Guera prefecture)	SSA	Low income	Unclear	All ages	Cross-sectional study	Clinic	351 (48%)	Leprosy	Clinical diagnosis	Footwear coverage; treatment completion rate	-	45	73	High: unclear measure of access; potential for selection bias
Suman et al. (2015) [65]	India (West Bengal)	SA	Low-middle income	Both	All ages	Cross-sectional study	Population	43,999 (1.3%)	Arthritis	Self-report (bespoke tool)	Care sought from: qualified provider (private), qualified (public)	4; 3	-	-	Medium: unreliable measure of disability
Tan et al. (2015) [28]	Malaysia (Penang)	EAP	Upper-middle	Unclear	Children	Cross-sectional study	Registry	305 (100%)	Physical impairment (unspecified)	Registry	Met need for: Mobility aid (e.g., wheelchair); Physiotherapy	59	44	-	Medium: low response rate
Wanaratwichit et al. (2008) [66]	Thailand (Phrae, Sukhothai, Chiang Rai)	EAP	Low-middle income	Unclear	Adults	Cross-sectional study	Population	406 (100%)	Physical impairment (unspecified)	Unclear	Proportion who have access to equipment; proportion who have access to physical rehabilitation	67	55	-	Medium: measure of disability unclear
Zongjie et al. (2007) [67]	China (Xincheng, Xuanwu, Beijing)	EAP	Low-middle income	Unclear	All ages	Cross-sectional study	Population, registry	460 (100%)	Various conditions	Registry	Received rehabilitation in the past 3 months	27	-	-	Medium: unclear means of assessing access and disability

SSA: sub-Saharan Africa, LA: Latin America, SA: South Asia, EAP: East Asia & Pacific, ME: Middle East; EU: Europe.

**Table 6:** Results of vision specific services.

Study Author, Year	Country	World Bank Region	Country Income Group	Locality	Age	Type of Study	Participant Source	N (D%)	Method of Assessment	Outcome	Proportion Covered by Type of Rehabilitation %			Risk of Bias
											Medical Rehabilitation	Assistive Device	Adherence	
Ahmad et al. (2015) [68]	Pakistan (Karachi)	SA	Low-middle income	Unclear	Older adults	Cross-sectional study	Population	638 (24%)	Visual acuity assessment; self-reported eye/vision problem	Ever sought treatment (blind; moderate visual impairment; severe visual impairment)	63; 50; 40	-	-	Low
Bernabe-Ortiz et al. (2016)	Peru (Morropon)	LA	Upper-middle	Semi-urban	All ages	Cross-sectional study	Population	798,308 (5%)	Washington Group short set	Coverage: Magnifying glasses	-	33	-	Medium: low response rate
Brian et al. (2012) [69]	Fiji (National)	EAP	Upper-middle	Both	Older adults	Cross-sectional study	Population	1381 (93%)	Visual acuity assessment and self-report	Consulted a provider (blind; low vision)	62; 53	-	-	Low
Devendra et al. (2013) [19]	Malawi (Lilongwe)	SSA	Low income	Unclear	Children	Case control study	Clinic	592 (50%)	WHO ten questions	Proportion of children who attended eye clinic of those in need	57	-	-	Low
Fletcher et al. (1999) [70]	India (Maduari)	SA	Low income	Rural	Adults	Cross-sectional study	Population	1039 (34%)	Visual acuity assessment	Attendance at camps for people identified as having need	7	-	-	Low
Kovai et al. (2007) [71]	India (Andhra Pradesh)	SA	Low-middle income	Rural	Adults	Cross-sectional study	Population	5573 (22%)	Visual acuity assessment	Sought treatment	31	-	-	Low
Kuper et al. (2016) [20]	Tanzania (Mbeya, Tanga, Lindi)	SSA	Low income	Both	All ages	Case control study	Population	254 (50%)	Washington Group short set	Coverage of: White cane; guide	-	18; 50	-	Low
Lee et al. (2013) [72]	Timor Leste (12 districts)	EAP	Low-middle income	Both	Older adults	Cross-sectional study	Population	2014 (93%)	Visual acuity assessment	Consulted care provider about vision problem: low vision/blindness; self-reported problem	25;26	-	-	Low
Maart et al. (2013) [21]	South Africa (Cape Town)	SSA	Upper-middle	Urban	All ages	Cross-sectional study	Population	151 (100%)	Washington Group short set	Proportion needing medical rehabilitation that received	57	-	-	Low
Mactaggart et al. (2015) [22]	Cameroon (Fundong Health District)	SSA	Low-middle income	Unclear	All ages	Case control study	Population	703 (61%)	Washington Group extended set	Coverage of: Magnifying glasses; white cane	-	15; 33	-	Low
	India (Mahbabnagar)	SA	Low-middle income					845 (60%)			-	46; 0	-	Low
Mahande et al. (2007) [73]	Tanzania (Hai)	SSA	Low income	Rural	Older adults	Cohort study	Population	163 (56%)	Visual acuity assessment	Trichiasis surgery uptake (visual impairment; blind)	47; 41	-	-	Medium: small sample size, response rate unclear
Malta et al. (2016) [23]	Brazil (National)	LA	Upper-middle	Both	All ages	Cross-sectional study	Population	204,000 (NS)	Self-report (bespoke tool)	Attendance at rehabilitation services	5	-	-	Low
Nesbitt et al. (2012) [24]	Bangladesh (Natore, Sirajgani)	SA	Low income	Both	Children	Key informant method initially; then prospective cohort study	Population	1308 (100%)	Clinical examination	Took up referral	-	-	31	Low
Palagyi et al. (2008) [74]	Timor Leste (Dili, Bobonaro)	EAP	Low-middle income	Both	Older adults	Cross-sectional study	Population	1414 (23%)	Visual acuity assessment	Sought treatment from Western Style health services	29	-	-	Low
Raban et al. (2010) [37]	India (Assam, Karnataka, Maharashtra, Rajasthan, Uttar Pradesh, West Bengal)	SA	Low-middle income	Both	Adults	Retrospective study	Population	9994 (NS)	Self-report (validated)	Treatment coverage	21	-	-	Medium: unreliable measure of disability
Tan et al. (2015) [28]	Malaysia (Penang)	EAP	Upper-middle	Unclear	Children	Cross-sectional study	Registry	305 (100%)	Registry	Met need for: Vision aids; Vision related services	52	47	-	Medium: low response rate; unclear means of assessing disability
Udeh et al. (2014) [75]	Nigeria (Enugu state)	SSA	Low income	Unclear	All ages	Cross-sectional study	Population	153 (100%)	Recruited through community leaders	Previous eye check; Used low vision device	82	0	-	High: unclear response rate; unclear measure of access

SSA: sub-Saharan Africa, LA: Latin America, SA: South Asia, EAP: East Asia & Pacific, ME: Middle East; EU: Europe.

**Table 7:** Access to any rehabilitation.

Study Author, Year	Country	World Bank Region	Country Income Group	Locality	Age	Type of Study	Participant Source	Sample Size	Means of Assessing Disability	Outcome	Proportion Covered by Type of Rehabilitation (%)			Risk of Bias
											General Rehab	Assistive Device	Adherence	
Bernabe-Ortiz et al. (2016) [17]	Peru (National)	LA	Upper-middle	Urban	All ages	Cross-sectional study	Population	798,608 (5%)	Washington Group short set	Any access to a range of rehabilitation services	11			Low
Bernabe-Ortiz et al. (2016) [76]	Peru (Morropon)	LA	Upper-middle	Semi-urban	All ages	Cross-sectional study (with nested case control)	Population	3684 (8%)	Washington Group short set	Proportion using rehabilitation now among those in need	5			Medium: low response rate
Borker et al. (2012) [77]	India (Goa)	SA	Low-middle income	Rural	Not presented	Cross-sectional study	Population	936 families (18%)	Bespoke tool/clinical examination	Use of rehabilitation care	24			High: unclear measure of disability, no response rate reported
Danquah et al. (2015) [18]	Haiti (Port-au-Prince)	LA	Low income	Urban	All ages	Case control study	Population	376 (50%)	Washington Group short set	Met need for specialist health care; medical rehabilitation; specialist advice	32; 49; 23	18		Low
Devendra et al. (2013) [19]	Malawi (Lilongwe)	SSA	Low income	Unclear	Children	Case control study	Clinic	592 (50%)	WHO ten questions	Access to: rehabilitation services, assistive devices	33	5		Low
Eide et al. (2003) [78]	Zimbabwe (National)	SSA	Low income	Both	All ages	Cross-sectional study	Population	1972 (100%)	Self-report (bespoke tool)	Received rehabilitation; assistive devices	55	36		Low
Loeb et al. (2004) [43]	Malawi (National)	SSA	Low income	Both	All ages	Cross-sectional study	Population	1574 (100%)	Self-report (bespoke tool)	Received rehabilitation; assistive devices	24	18		Low
Eide et al. (2003) [79]	Namibia (National)	SSA	Low-middle	Both	All ages	Cross-sectional study	Population	2528 (100%)	Self-report (bespoke tool)	Received rehabilitation; assistive devices	26	17		Low
Eide et al. (2006) [44]	Zambia (National)	SSA	Low income	Both	All ages	Cross-sectional study	Population	2865 (100%)	Washington Group short set	Received rehabilitation; assistive devices	37	18		Low
Eide et al. (2009) [80]	Mozambique (National)	SSA	Low income	Both	All ages	Cross-sectional study	Population	666 (100%)	Washington Group short set	Received rehabilitation; assistive devices	38	18		Low
Eide et al. (2011) [81]	Swaziland (National)	SSA	Low-middle	Both	All ages	Cross-sectional study	Population	866 (100%)	Washington Group short set	Received rehabilitation; assistive devices	31	32		Low
Eide et al. (2016) [82]	Nepal (National)	SA	Low income	Both	All ages	Cross-sectional study	Population	2123 (100%)	Washington Group short set	Received rehabilitation; assistive devices	22	22		Low
Eide et al. (2016) [83]	Botswana (National)	SSA	Upper-middle	Both	All ages	Cross-sectional study	Population	2123 (100%)	Washington Group short set	Received rehabilitation; assistive devices	33	34		Low
Hamdan et at. (2009) [84]	Palestine (Tulkarm, Qualqilia)	ME	Low-middle	Rural	All ages	Cross-sectional study	Population	806 (100%)	Clinical examination	Use of equipment		19		Low
Hosain et al. (1998) [85]	Bangladesh (Maniramore Thana, Jessore district)	SA	Low income	Rural	All ages	Cross-sectional study	Population	1906 (8%)	Head of household report	Sought treatment from qualified provider	34			Medium: unreliable measure of disability
Kisioglu et al. (2003) [86]	Turkey (Isparta)	EU	Low-middle	Both	All ages	Cross-sectional study	Population	3500 (5%)	Self-report (bespoke tool)	Receipt of rehabilitation	5			High: unreliable measure of disability; unclear response rate
Kuper et al. (2015) [87]	Kenya (Turkana)	SSA	Low income	Unclear	Children	Case control study	Population	807 (39%)	Washington Group short set	Receipt of rehabilitation	15			Low
Kuper et al. (2016) [20]	Tanzania (Mbeya, Tanga, Lindi)	SSA	Low income	Both	All ages	Case control study	Population	254 (50%)	Washington Group short set	Coverage of rehabilitation services; specialist health services; assistive devices	20; 5	33		Low
Maart et al. (2013) [21]	South Africa (Cape Town)	SSA	Upper-middle	Urban	All ages	Cross-sectional study	Population	151 (100%)	Washington Group short set	Medical rehabilitation; assistive device	71	66		Low
Mactaggart et al. (2015) [22]	India (Mahbabnagar)	SA	Low-middle income	Unclear	All ages	Case control study	Population	703 (61%)	Washington Group extended set	Met need for medical rehabilitation; assistive devices	61	48		Low
	Cameroon (Fundong Health District)	SSA	Low-middle income					845 (60%)			76	44		
Marella et al. (2014) [88]	Fiji (not specified)	EAP	Upper-middle	Both	Adults	Case control study	Population	101 (50%)	Rapid Assessment of Disability	Access to rehabilitation; access to assistive devices	45	35		Low
	Bangladesh (Bogra)	SA	Low income					195 (50%)			7	12		
Marella et al. (2016) [89]	Philippines (Quezon, Liago City)	EAP	Low-middle income	Both	Adults	Case control study	Population	204,000 (6%)	Rapid Assessment of Disability	Access to rehabilitation; Access to assistive devices	70	46		Low
Nesbitt et al. (2012) [24]	Bangladesh (Natore, Sirajgani)	SA	Low income	Both	Adults	Prospective cohort study	Population	1308 (100%)	Clinical examination	Uptake of referral			48	Low
Nualnetr et al. (2012) [90]	Thailand (Non Bon, Kosum Phisai, Maha Sarakham)	EAP	Low-middle income	Rural	Not specified	Cross-sectional study	Registry	99 (99; 100%)	Not specified	Assistive device received and appropriate		33	-	Low
Padmamohan et al. (2009) [26]	India (Kerala)	SA	Low-middle income	Rural	Children	Cross-sectional study	Population	98 (100%)	Community health workers assessment	Use of rehabilitation treatment	48			Medium: small sample size, method of disability assessment unreliable
Pongprapai et al. (1996) [91]	Thailand (Nongjik)	EAP	Low-middle	Unclear	Children	Cross-sectional study	Population	53 (100%)	Bespoke questionnaire and clinical examination	Sought treatment for child’s condition	62			Medium: unclear measure of disability; unclear response rate
Souza et al. (2012) [92]	Brazil (Bahia)	LA	Upper-middle	Urban	All ages	Cross-sectional study	Population	235 (100%)	Self-report (bespoke tool)	Ever received treatment	80			Medium: unclear measure of disability
Tan et al. (2015) [28]	Malaysia (Penang)	EAP	Upper-middle	Unclear	Children	Cross-sectional study	Registry	305 (100%)	Registry	Met need for services (specialist doctor; therapy; assistive device)	76			Medium: low response rate

SSA: sub-Saharan Africa, LA: Latin America, SA: South Asia, EAP: East Asia & Pacific, ME: Middle East; EU: Europe.

**Table 8:** Barriers to accessing rehabilitation reported across studies.

Barrier	Reference
Geographic accessibility	
Distance to service	[19,21,26,28,31,47,69,71,72,74,93]
Transport problems	[18,19,21,28,31,69,72,74,77,84,89,94]
Nobody to accompany	[28,69,71,72,74,77,93]
Affordability	
Unable to afford services	[18,19,20,21,22,26,27,31,47,58,62,67,71,72,74,77,84,89]
Unable to afford treatment	[19,47,60,70,75,93]
No insurance	[47]
Acceptability	
Do not know where to go for treatment	[27,28,31,47,48,69,71,72,74,93]
Have not heard about service	[75]
Thought nothing could be done	[31,48,69,70,71,72,74]
Lack of perceived need	[20,31,47,48,69,70,71,72,74,95]
Family do not perceive need	[71]
Fear of seeking care	[31,69,70,71,72,74]
No time/other priorities	[28,47,69,70,71,72,74,84,93]
Other medical problems	[60,71]
Shame	[31,95]
Lack of trust in healthcare providers keeping confidentiality	[31]
Availability	
Waiting time at the clinic	[31,74,77]
Not availability of drugs, services	[21,28,60,75,84,93]
Quality	
Discrimination/poor treatment from health provider	[19,21,28,31,47,69]
Poor relationship with provider	[70,71,95]
Provider refused care	[28,84]
Communication barrier	[21]
Provider lacks skills	[28,67]

## Supplementary Materials

The following are available online at http://www.mdpi.com/1660-4601/15/10/2165/s1, Table S1: EMBASE search strategy.
