# Supplementary material for: A Systematic Review of Access to Rehabilitation for People with Disabilities in Low- and Middle-Income Countries
Source: Int J Environ Res Public Health. 2018 Oct 2;15(10):2165. doi: 10.3390/ijerph15102165 (PMC6210163; doi:10.3390/ijerph15102165)
Supplement: Supplementary file 1 [file ijerph-15-02165-s001.pdf]

**Table S1**. EMBASE search strategy.

| # | Searches | Results | Annotations |
|---|---|---|---|
| 1 | Developing.mp. [mp=title, abstract, heading word, drug trade name, original title, device manufacturer, drug manufacturer, device trade name, keyword, floating subheading word] | 598110 | |
| 2 | low-income.mp. [mp=title, abstract, heading word, drug trade name, original title, device manufacturer, drug manufacturer, device trade name, keyword, floating subheading word] | 30476 | |
| 3 | low income.mp. [mp=title, abstract, heading word, drug trade name, original title, device manufacturer, drug manufacturer, device trade name, keyword, floating subheading word] | 30476 | |
| 4 | middle-income.mp. [mp=title, abstract, heading word, drug trade name, original title, device manufacturer, drug manufacturer, device trade name, keyword, floating subheading word] | 12451 | |
| 5 | middle income.mp. [mp=title, abstract, heading word, drug trade name, original title, device manufacturer, drug manufacturer, device trade name, keyword, floating subheading word] | 12451 | |
| 6 | (low and middle income).mp. [mp=title, abstract, heading word, drug trade name, original title, device manufacturer, drug manufacturer, device trade name, keyword, floating subheading word] | 10722 | |
| 7 | less-developed.mp. [mp=title, abstract, heading word, drug trade name, original title, device manufacturer, drug manufacturer, device trade name, keyword, floating subheading word] | 3357 | |
| 8 | (low- and middle-income).mp. [mp=title, abstract, heading word, drug trade name, original title, device manufacturer, drug manufacturer, device trade name, keyword, floating subheading word] | 10722 | |
| 9 | less developed.mp. [mp=title, abstract, heading word, drug trade name, original title, device manufacturer, drug manufacturer, device trade name, keyword, floating subheading word] | 3357 | |
| 10 | under developed.mp. [mp=title, abstract, heading word, drug trade name, original title, device manufacturer, drug manufacturer, device trade name, keyword, floating subheading word] | 685 | |
| 11 | underdeveloped.mp. [mp=title, abstract, heading word, drug trade name, original title, device manufacturer, drug manufacturer, device trade name, keyword, floating subheading word] | 5363 | |
| 12 | third-world.mp. [mp=title, abstract, heading word, drug trade name, original title, device manufacturer, drug manufacturer, device trade name, keyword, floating subheading word] | 3405 | |
| 13 | third world.mp. [mp=title, abstract, heading word, drug trade name, original title, device manufacturer, drug manufacturer, device trade name, keyword, floating subheading word] | 3405 | |
| 14 | 1 or 2 or 3 or 4 or 5 or 6 or 7 or 8 or 9 or 10 or 11 or 12 or 13 | 640660 | |
| 15 | ((Developing or low-income or low income or middle-income or middle income or (low and middle income) or less-developed or (low- and middle-income) or less developed or under developed or underdeveloped or third-world or third world) adj5 (countr* or nation* or world or econom*)).mp. | 146531 | |
| 16 | exp Developing Countries/ | 87090 | |
| 17 | (LIC or LICs or MIC or MICs or LMIC or LMICs or LAMIC or LAMICs or LAMI countr*).mp. [mp=title, abstract, heading word, drug trade name, original title, device manufacturer, drug manufacturer, device trade name, keyword, floating subheading word] | 56705 | |
| 18 | (Transitional countr* or Transitional econom* or Transition countr* or Transition econom*).mp. [mp=title, abstract, heading word, drug trade name, original title, device manufacturer, drug manufacturer, device trade name, keyword, floating subheading word] | 437 | |
| 19 | (Africa or Asia or Caribbean or West Indies or Latin America or Central America or South America).mp. [mp=title, abstract, heading word, drug trade name, original title, device manufacturer, drug manufacturer, device trade name, keyword, floating subheading word] | 300779 | |
| 20 | exp Africa South of the Sahara/ or exp Africa/ or exp Asia, Central/ or exp Asia, South East/ or exp Asia, Western/ or exp Latin America/ or exp Caribbean/ or exp Central America/ or exp South America/ | 1272648 | |
| 21 | (Afghanistan or Albania or Algeria or American Samoa or Angola or Antigua or Barbuda or Argentina or Armenia or Azerbaijan or Bangladesh or Belarus or Byelarus or Byelorussia or Belorussia or Belize or Benin or Bhutan or Bolivia or Bosnia or Herzegovina or Hercegovina or Bosnia-Herzegovina or Bosnia-Hercegovina or Botswana or Brazil or Brasil or Bulgaria or Burkina or Upper Volta or Burundi or Urundi or Cambodia or Republic of Kampuchea or Cameroon or Cameroons or Cape Verde or Central African Republic or Chad or Chile or China or Colombia or Comoros or Comoro Islands or Comores or Congo or DRC or Zaire or Costa Rica or Cote d'Ivoire or Ivory Coast or Cuba or Djibouti or Obock or French Somaliland or Dominica or Dominican Republic or Ecuador or Egypt or United Arab Republic or El Salvador or Eritrea or Ethiopia or Fiji or Gabon or Gabonese Republic or Gambia or Georgia or | 1608277 | |

| | | |
|---|---|---|
| | Ghana or Gold Coast or Grenada or Guatemala or Guinea or Guinea-Bissau or Guiana or Guyana or Haiti or Honduras or India or Indonesia or Iran or Iraq or Jamaica or Jordan or Kazakhstan or Kenya or Kiribati or Republic of Korea or North Korea or DPRK or Kosovo or Kyrgyzstan or Kirghizstan or Kirgizstan or Kirghizia or Kirgizia or Kyrgyz or Kirghiz or Kyrgyz Republic or Lao or Laos or Latvia or Lebanon or Lesotho or Basutoland or Liberia or Libya or Lithuania or Macedonia or Madagascar or Malagasy Republic or Malawi or Nyasaland or Malaysia or Malaya or Malay or Maldives or Mali or Marshall Islands or Mauritania or Mauritius or Mayotte or Mexico or Micronesia or Moldova or Moldovia or Mongolia or Montenegro or Morocco or Mozambique or Myanmar or Burma or Namibia or Nepal or Nicaragua or Niger or Nigeria or Pakistan or Palau or Palestine or Panama or Papua New Guinea or Paraguay or Peru or Philippines or Romania or Rumania or Roumania or Russia or Russian Federation or USSR or Soviet Union or Union of Soviet Socialist Republics or Rwanda or Ruanda-Urundi or Samoa or Samoan Islands or Sao Tome or Principe or Senegal or Serbia or Montenegro or Yugoslavia or Seychelles or Sierra Leone or Solomon Islands or Somalia or South Africa or Sri Lanka or Ceylon or Saint Kitts or St Kitts or Saint Christopher Island or Nevis or Saint Lucia or St Lucia or Saint Vincent or St Vincent or Grenadines or Sudan or Suriname or Surinam or Swaziland or Syria or Syrian Arab Republic or Tajikistan or Tadzhikistan or Tadjikistan or Tanzania or Thailand or Timor-Leste or East Timor or Togo or Togolese Republic or Tonga or Tunisia or Turkey or Turkmenistan or Turkmenia or Tuvalu or Uganda or Ukraine or Uruguay or Uzbekistan or Vanuatu or New Hebrides or Venezuela or Vietnam or Viet Nam or West Bank or Gaza or Yemen or Zambia or Zimbabwe or Rhodesia).mp. [mp=title, abstract, heading word, drug trade name, original title, device manufacturer, drug manufacturer, device trade name, keyword, floating subheading word] | |
| 22 | 15 or 16 or 17 or 18 or 19 or 20 or 21 | 2149386 |
| 23 | ((person* with disabilit* or people with disabilit* or (disable* or Disabilit* or Handicap*)) adj5 (person* or people)).mp. [mp=title, abstract, heading word, drug trade name, original title, device manufacturer, drug manufacturer, device trade name, keyword, floating subheading word] | 51530 |
| 24 | (Physical* adj5 (impair* or deficienc* or disable* or disabili* or handicap*)).mp. [mp=title, abstract, heading word, drug trade name, original title, device manufacturer, drug manufacturer, device trade name, keyword, floating subheading word] | 38130 |
| 25 | (Cerebral pals* or Spina bifida or Muscular dystroph* or Arthriti* or Osteogenesis imperfecta or Musculoskeletal abnormalit* or Musculo-skeletal abnormalit* or Muscular abnormalit* or Skeletal abnormalit* or Limb abnormalit* or Amputation* or Clubfoot or Poliomyeliti* or Paraplegi* or Paralys* or Paralyz* or Hemiplegi*).mp. [mp=title, abstract, heading word, drug trade name, original title, device manufacturer, drug manufacturer, device trade name, keyword, floating subheading word] | 687454 |
| 26 | ((Hearing or Acoustic or Ear$3) adj5 (loss* or impair* or deficienc* or disable* or disabili* or handicap*)).mp. [mp=title, abstract, heading word, drug trade name, original title, device manufacturer, drug manufacturer, device trade name, keyword, floating subheading word] | 137842 |
| 27 | ((Visual* or Vision or Eye$3) adj5 (loss* or impair* or deficienc* or disable* or disabili* or handicap*)).mp. [mp=title, abstract, heading word, drug trade name, original title, device manufacturer, drug manufacturer, device trade name, keyword, floating subheading word] | 86190 |
| 28 | (Deaf* or Blind*).mp. [mp=title, abstract, heading word, drug trade name, original title, device manufacturer, drug manufacturer, device trade name, keyword, floating subheading word] | 480529 |
| 29 | exp Hearing impairment/ or exp vision disorders/ or exp Deafness/ or exp Blindness/ | 294763 |
| 30 | (Schizophreni* or Psychosis or Psychoses or Psychotic Disorder* or Schizoaffective Disorder* or Schizophreniform Disorder* or Dementia* or Alzheimer*).mp. [mp=title, abstract, heading word, drug trade name, original title, device manufacturer, drug manufacturer, device trade name, keyword, floating subheading word] | 540291 |
| 31 | exp Schizophrenia/ | 173735 |
| 32 | exp Dementia/ or exp Alzheimer disease/ | 294153 |
| 33 | 31 or 32 | 459369 |
| 34 | ((Intellectual* or Mental* or Psychological* or Developmental) adj5 (impair* or retard* or deficienc* or disable* or disabili* or handicap* or ill?6)).mp. [mp=title, abstract, heading word, drug trade name, original title, device manufacturer, drug manufacturer, device trade name, keyword, floating subheading word] | 130687 |
| 35 | ((communication or language or speech or learning) adj5 disorder*).mp. [mp=title, abstract, heading word, drug trade name, original title, device manufacturer, drug manufacturer, device trade name, keyword, floating subheading word] | 67030 |
| 36 | exp Disabled Children/ | 8489 |

| | | |
|---|---|---|
| 37 | exp Learning Disorders/ | 32681 |
| 38 | exp Mentally Disabled Persons/ | 184 |
| 39 | 36 or 37 or 38 | 41077 |
| 40 | 23 or 24 or 25 or 26 or 27 or 28 or 29 or 30 or 33 or 34 or 35 or 39 | 2167263 |
| 41 | 40 not (disability adjusted life year* or DALY).mp. [mp=title, abstract, heading word, drug trade name, original title, device manufacturer, drug manufacturer, device trade name, keyword, floating subheading word] | 2166778 |
| 42 | 41 not (double blind* or single blind* or blinded study).mp. [mp=title, abstract, heading word, drug trade name, original title, device manufacturer, drug manufacturer, device trade name, keyword, floating subheading word] | 1909397 |
| 43 | exp Insurance, Health/ | 221903 |
| 44 | exp Palliative Care/ | 89105 |
| 45 | exp Preventive Health Services/ | 25024 |
| 46 | exp Child Health Services/ | 85873 |
| 47 | exp Maternal Health Services/ | 307 |
| 48 | exp Primary Health Care/ | 135154 |
| 49 | exp Health Services/ | 4520626 |
| 50 | exp Health Promotion/ | 82697 |
| 51 | exp Immunization/ | 272930 |
| 52 | exp Immunization Programs/ | 25024 |
| 53 | exp Rehabilitation/ | 330424 |
| 54 | exp Family Planning Services/ | 36282 |
| 55 | exp Reproductive Health Services/ | 4520626 |
| 56 | exp Mass Screening/ | 196915 |
| 57 | exp Prenatal Care/ | 132573 |
| 58 | exp Breast Feeding/ | 43466 |
| 59 | exp Smoking Cessation/ | 47753 |
| 60 | exp Anti-HIV Agents/ | 137207 |
| 61 | exp Insecticide-Treated Bednets/ | 303 |
| 62 | exp Fluid Therapy/ | 80769 |
| 63 | exp Hand Disinfection/ | 86 |
| 64 | (access* or equal* or inequal* or barrier*).mp. [mp=title, abstract, heading word, drug trade name, original title, device manufacturer, drug manufacturer, device trade name, keyword, floating subheading word] | 1196730 |
| 65 | exp Papillomavirus Vaccines/ | 9990 |
| 66 | exp Hepatitis B Vaccines/ | 17524 |
| 67 | 47 or 54 or 55 or 57 or 58 or 59 or 60 or 61 or 62 or 63 or 65 or 66 | 4831428 |
| 68 | 43 or 44 or 45 or 46 or 48 or 49 or 50 or 51 or 52 or 53 | 4938817 |
| 69 | 64 and 68 | 310147 |
| 70 | 22 and 42 and 69 | 3229 |
| 71 | 67 or 68 | 5228079 |
| 72 | 64 and 71 | 324011 |
| 73 | 22 and 42 and 72 | 3321 |
| 74 | exp Tuberculosis/ | 234046 |
| 75 | (prevent* or treat* or diagn*).mp. [mp=title, abstract, heading word, drug trade name, original title, device manufacturer, drug manufacturer, device trade name, keyword, floating subheading word] | 12400328 |
| 76 | 74 and 75 | 150547 |
| 77 | exp Antihypertensive Agents/ | 653359 |
| 78 | 67 or 76 or 77 | 5482294 |
| 79 | 68 or 78 | 5869969 |
| 80 | 64 and 79 | 341918 |
| 81 | 22 and 42 and 80 | 3428 |
| 82 | from 73 keep 1-1039 | 1039 |

| | | |
|---|---|---|
| 83 | from 81 keep 1-1065 | 1065 |
| 84 | 56 or 68 | 5025074 |
| 85 | 84 or health*.mp. [mp=title, abstract, heading word, drug trade name, original title, device manufacturer, drug manufacturer, device trade name, keyword, floating subheading word] | 7384455 |
| 86 | 78 or 85 | 8209053 |
| 87 | 64 and 86 | 464932 |
| 88 | 22 and 42 and 87 | 4691 |
